# Association between Vaginal Micro-environment Disorder and Cervical Intraepithelial Neoplasia in a Community Based Population in China

**DOI:** 10.7150/jca.35022

**Published:** 2020-01-01

**Authors:** Li Li, Ling Ding, Tao Gao, Yuanjing Lyu, Ming Wang, Li Song, Xiaoxue Li, Wen Gao, Yang Han, Haixia Jia, Jintao Wang

**Affiliations:** 1 Department of Epidemiology, School of Public Health, Shanxi Medical University, Taiyuan, China.; 2 Department of Preventive Medicine, Robert H. Lurie Comprehensive Cancer Center, Feinberg School of Medicine, Northwestern University, Chicago 60611, USA.

**Keywords:** cervical intraepithelial neoplasia, HPV16, vaginal micro-environment factors

## Abstract

There are other factors that contribute to cervical carcinogenesis except HPV infection. This study aimed to investigate the association between vaginal micro-environment factors, including H_2_O_2_, vaginal PH value, vagina cleanness, β-glucuronidase, coagulase, neuraminidase and leukocyte esterase and cervical intraeipithelial neoplasia (CIN). In total 1019 participants, including 623 normal cervical (NC) women, 303 patients with low-grade cervical intraepithelial neoplasia (CIN1) and 93 patients with high-grade cervical intraepithelial neoplasia (CIN2/3), were enrolled into the study. HPV genotyping was detected by flow-through hybridization and gene chip. Vaginal H_2_O_2_, β-glucuronidase, coagulase, neuraminidase and leukocyte esterase were detected by Aerobic Vaginitis (AV) / Bacterial Vaginal Disease (BV) Five Joint Test Kit. Vaginal PH was measured on the glass slide after microscopy, using color strips with a PH range of 3.8-5.4. Vagina cleanness was determined according to the National Clinical Laboratory Practice Guideline. *χ^2^*test and Logistic regression were operated using SPSS 22.0 software. Our results showed that HPV16 infection rate and the abnormal rates of H_2_O_2_, PH, vagina cleanness, β-glucuronidase or neuraminidase increased gradually along with the severity of CIN (*P*<0.05). Abnormities of H_2_O_2_, cleanness, β-glucuronidase and neuraminidase were risk factors for CIN regardless of HPV16 infection, furthermore, abnormities of PH value, leukocyte esterase could also increase the risk of CIN in HPV16 positive group. In addition, women with abnormal vaginal micro-environment factors in HPV16 positive group had a significantly higher risk of developing CIN than HPV16 negative group. The results from generalized multifactor dimensionality reduction (GMDR) model showed that there was interaction effect with abnormities of vagina cleanness, H_2_O_2_, β-glucuronidase and neuraminidase on CIN2/3 in HPV16 negative group, while, there was interaction effect with abnormities of vagina cleanness, β-glucuronidase and neuraminidase on CIN1 and with abnormities of vagina cleanness, PH, H_2_O_2_, β-glucuronidase, neuraminidase and leukocyte esterase on CIN2/3 in HPV16 positive group. Our results suggested that vaginal micro-environment disorder could increase the risk of CIN, especially, the abnormality of H_2_O_2_, cleanness, β-glucuronidase and neuraminidase. There were interaction effects with abnormities of H_2_O_2_, vagina cleanness, β-glucuronidase and neuraminidase on CIN whether HPV16 was infected or not.

## Introduction

Cervical cancer is one of the most common gynecological malignancies worldwide with an estimated 570,000 cases and 311,000 deaths in 2018 1. The causal role of high-risk human papillomavirus (HR-HPV) in cervical intraepithelial neoplasia (CIN) has been approved by various studies 2, 3. Especially, HPV16 is by far the most common type of carcinogenic globally, with the positivity rate of over 60% in cervical cancer patients 4. The majority of HPV infections are transient with subclinical or asymptomatic appearance 5, 6, only a small proportion of women infected with the virus develop clinically significant pre-invasive lesions and cervical cancer. As a precancerous lesion of cervical cancer, it takes an average of 8 years for CIN to develop into cervical cancer. Therefore, it is of great significance to explore the main risk factors of CIN for blocking the occurrence of cervical cancer.

In recent years, the researches on cancer or chronic disease focused on the relationship between intestinal micro-ecology 7, 8 and oral micro-ecology 9. The association between vaginal micro-ecology and cervical cancer had also been concerned. But there is no clear definition of guiding vaginal micro-environment disorder up to now. Vaginal PH value, H_2_O_2_, vagina cleanness, β-glucuronidase, coagulase, neuraminidase and leukocyte esterase are important evaluation index to evaluate the vaginal micro-ecology in clinical practice 10, 11, which could comprehensively reflect vaginal micro-ecology from vaginal acidity and alkalinity, Lactobacillus function, microbial metabolites and inflammatory reaction, therefore, they are generally referred as vaginal micro-environmental factors. It was reported that increased vaginal PH could increase the risk of HPV infection by 30% 12. H_2_O_2_ can catalyze peroxidase to produce hypochlorite, which can prevent HPV virus from colonizing and growing in cervical epithelial cells 13. Poor cleanness may be related to bacterial vaginitis 14 and aerobic vaginitis 15. A case-control study found that neuraminidase abnormal rate was significantly higher in patients with bacterial vaginitis than in non-patients 16. Leukocyte esterase activity in patients with recurrent vaginal candidiasis and bacterial vaginitis was higher than that in normal women 17. Although these studies suggest that vaginal micro-environment factors may plays an important role in HPV infection and bacterial vaginitis, it is not clear about the potential function of vaginal micro-environment factors on CIN progression. This study aimed to explore the association between vaginal micro-environment factors and CIN in regard of HPV16 infection so as to provide new ideas for the further study of CIN.

## Materials and Methods

### Study population

**Figure 1:**
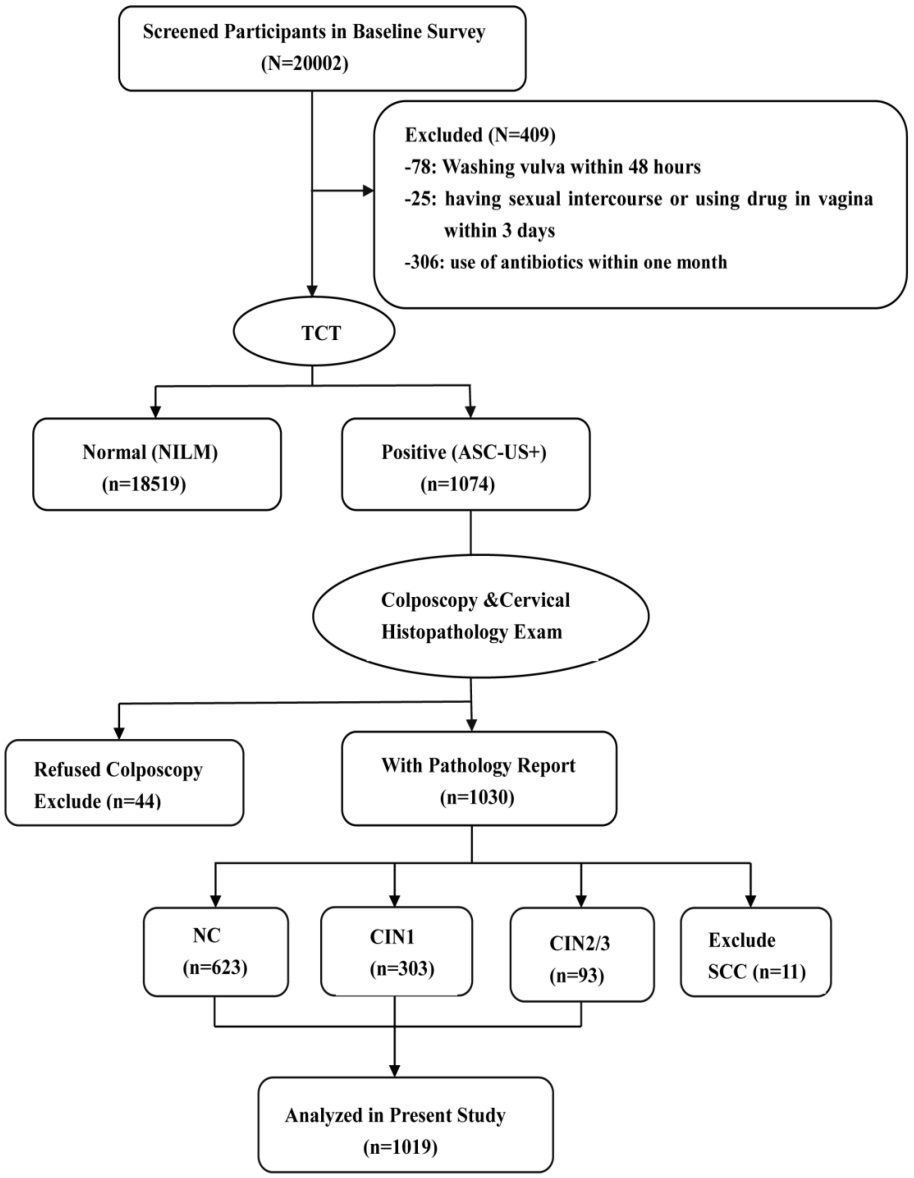
Flow chart of participant screening in the study. TCT, Thinprep Cytologic Test. ASC-US+, atypical squamous cells of undetermined significance or above. NILM, negative for intraepithelial lesion or malignancy. CIN, cervical intraepithelial neoplasia.

1019 participants were enrolled in the current study, including 623 participants with NC, 303 with CIN1, and 93 with CIN2/3. These participants came from the community cohort which was established during June 2014 to September 2014 and included around 20000 women in Jiexiu, Shanxi, China. The participants were considered eligible according to the following inclusion criteria: married, aged 18-65 years, Han ethnicity, and resided in Jiexiu for at least 1 year and the exclusion criteria included the following: washed vulva within 48 hours, had sexual intercourse or used drug in vagina within the last 3 days, used of antibiotics within one month. A cytobrush was used in the gynecological examination to collect exfoliative cells for TCT (Thinprep Cytologic Test) detection. Total 19593 women in the cohort completed TCT detection and 1074 women of them were diagnosed as atypical squamous cells of undetermined significance (ASC-US) and above. Among them, 44 women who refused to undergo colposcope and histopathologic examination and 11 women with cervical squamous cell carcinoma (SCC) were excluded (**Fig.1**). Informed consent was obtained from all individual participants and the study was approved by Shanxi Medical University Science Research Ethics Committee.

### Sample collection

Information about participants' socio- demographic characteristics, vaginal hygiene, history of gynecological disease, family history of cancer was collected using structured questionnaire. Cervical exfoliative cells were collected for HPV detection. Sterile cotton swab was used to collect secretions on the posterior fornix of vagina, and then put into a soft plastic container which contains 400μl dilution. Cervical tissues were collected using colposcopy directed biopsy for pathological test.

### HPV16 detection

The exfoliative cells were centrifuged for 15min at 4°C, 13000 rpm. Then HPV-DNA was extracted using DNA extraction kit (Hybribio, China). PCR-based flow-through hybridization and gene chip system (Hybribio, China) was used for HPV genotyping. This genotyping test could qualitatively detect 15 high-risk types: HPV16, 18, 31, 33, 35, 39, 45, 51, 52, 53, 56, 58, 59, 66, 68 and 6 low-risk types: HPV6, 11, 42, 43, 44, CP8304. Single or mixed HPV16 infection was defined as HPV16 infections in this study.

### Detection of vaginal PH and vagina cleanness

Vaginal PH value was measured using color strips with a PH range of 3.8-5.4. It was normal if PH was no more than 4.5 (PH≤4.5) and abnormal if PH>4.5. Vagina cleanness was judged strictly according to the National Clinical Laboratory Practice Guideline 18 standard: I~II was defined as normal, and III~IV as abnormal.

### Detection of bacterial enzymes in vagina

In accordance with the operation instructions of the Aerobic Vaginitis (AV) / Bacterial Vaginal Disease (BV) Five Joint Test Kit (Beijing ZhongSheng JinYu Diagnosis Technology Co, Ltd., Beijing, China.), a drop of sample (about 35 ml) was added into each well. After incubating the mixture for 10 minutes at 37°C, a drop of color development solution A and B were added to the neuraminidase well and the coagulase well respectively. According to the technicians' instruction, the results were interpreted. In this study, negative results were defined as normal and positive results as abnormal. Besides, the standard quality control products in the kit were used for quality control testing of each indicator to ensure the accuracy of the experimental results.

### Definition of vaginal micro-environment disorder

Currently, there is no clear definition of guiding vaginal micro-environment disorder. In our study, it was defined as any of the above vaginal micro-environment factors abnormalities, including vaginal PH value, H_2_O_2_, vagina cleanness, β-glucuronidase, coagulase, neuraminidase and leukocyte esterase, which are important biomarkers for evaluating vaginal micro-ecology in clinical practice 10, 11.

### Statistical analyses

Data analyses were performed with SPSS 22.0 statistical software. Count data were examined by Chi-square and trend Chi-square tests. Multinomial logistic regression model was used to estimate odds ratio (*OR*). And the interaction among multiple factors was analyzed by generalized multifactor dimensionality reduction (GMDR). Statistical significance was set at *α*=0.05.

## Results

### Socio-demographic characteristics and relevant factors

Our study showed that there were significant differences on occupation, passive smoking, frequency of vulva cleaning, frequency of underwear washing, washing vulva after sexual intercourse and contraception history between NC group, CIN1 group and CIN2/3 group (**Table 1**). There were no significant differences on age, education, marital status, age at menarche, gynecological history, numbers of pregnancy et al among the three groups (*P*>0.05).

### Related factors of cervical lesions

The first three principal components are selected since their eigenvalues are greater than 1 (**Table 2**). The latent variable FAC1 represented frequency of vulva cleaning and frequency of underwear washing. Latent variable FAC2 represented washing vulva after sexual intercourse and occupation. Latent variable FAC3 represented passive smoking and contraception history. Partial correlation KMO (Kaiser-Meyer- Olkin) is 0.658, which was suitable for factor analysis (**Table 3**).

### Association between HPV16 and CIN

**Figure 2:**
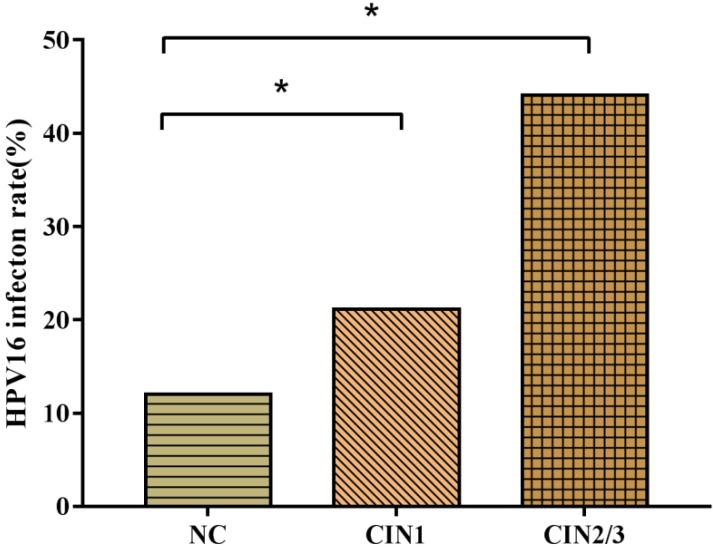
HPV16 infection rates in CIN. HPV16 infection rates ascended with the progression of cervical lesions (*χ^2^_trend_*=55.45, *P*<0.001). * There was a statistical difference in comparison to NC group adjusted by FAC1, FAC2 and FAC3 (*P*<0.05).

HPV16 infection rates in women with CIN1 (21.1%) and CIN2/3 (44.1%) were significantly higher than women with NC (12.0%). The probability of HPV16 positive individuals to develop CIN1 and CIN2/3 were respectively 1.97 and 5.71 times than HPV16 negative ones. With the progression of CIN, the infection rate of HPV16 (*χ^2^_trend_*=55.45, *P*<0.001) increased gradually (**Fig.2**).

### Association between vaginal micro-environment factors and CIN

**Figure 3:**
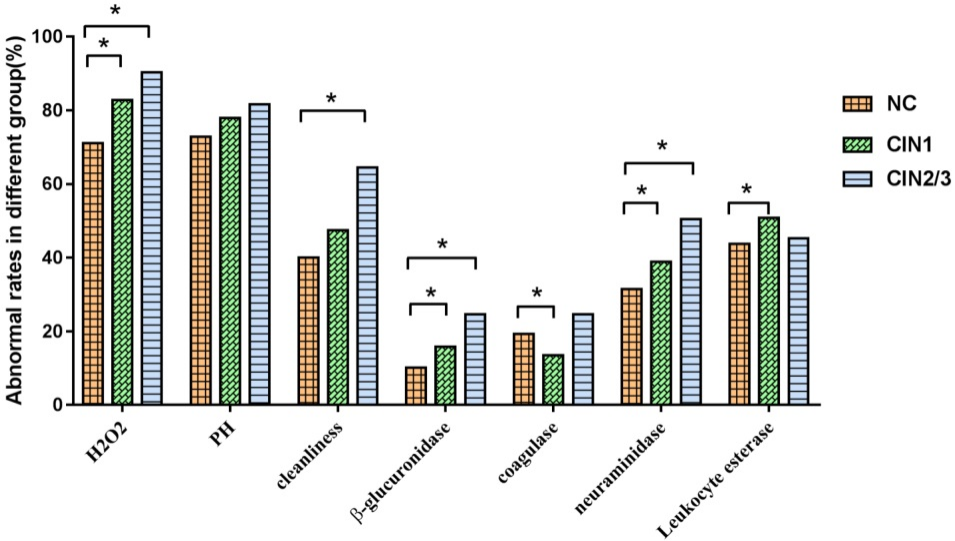
Association between vaginal micro-environment factors and CIN. *There was a statistical difference in comparison to NC group (*P*<0.05).

There were significant differences in the abnormal rates of H_2_O_2_ (*χ^2^*=26.60, *P*<0.001), vagina cleanness (*χ^2^*=21.02, *P*<0.001), β-glucuronidase (*χ^2^*=17.88, *P*<0.001), coagulase (*χ^2^*=7.61, *P*=0.022) and neuraminidase (*χ^2^*=15.21, *P*<0.001) in the groups of NC, CIN1, CIN2/3. The abnormal rates of H_2_O_2_, β-glucuronidase, neuraminidase and leukocyte esterase in CIN1 group were significantly higher than that in NC group, while the abnormal rates of H_2_O_2_, vagina cleanness, β-glucuronidase and neuraminidase in CIN2/3 group were significantly higher than in NC group. We found that the abnormal rates of H_2_O_2_, PH, vagina cleanness, β-glucuronidase and neuraminidase were gradually increased with the severity of cervical lesions (*P*<0.05), showing in **Fig.3**.

### Association between vaginal micro-environment factors and CIN in different HPV16 infection state

**Figure 4:**
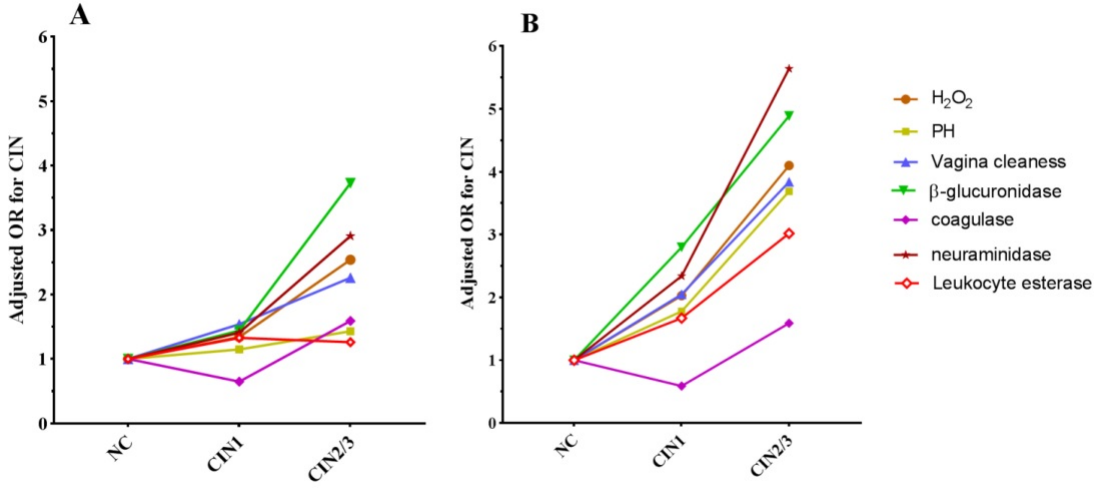
Trend of OR of vaginal micro-environment factors with the progress of cervical lesions in HPV16 negative group (A) and HPV16 positive group (B).

In the HPV16 negative group, H_2_O_2_, cleanness, β-glucuronidase and neuraminidase abnormities were risk factors for CIN2/3, and the abnormal rates increased with the severity of cervical lesions (**Table 4**). Among women with HPV16 infection, there were significant differences between the three groups in abnormal rates of H_2_O_2_, PH, cleanness, β-glucuronidase, coagulase, neuraminidase and leukocyte esterase, meanwhile, we observed that H_2_O_2_, cleanness, β-glucuronidase and neuraminidase abnormities were collective risk factors both in CIN1 and CIN2/3 group and abnormal PH and leukocyte esterase were also risk factors for CIN2/3 (**Table 4**). In addition, women with abnormal vaginal micro- environment factors in HPV16 positive group had a significantly higher risk on CIN than in HPV16 negative group (**Table 4 and Fig.4**).

### Interaction between vaginal micro-environment factors in CIN according to HPV16 infection

In the HPV16 negative group, there was interaction effect with abnormities of H_2_O_2_, vagina cleanness, β-glucuronidase and neuraminidase on CIN2/3. In the HPV16 positive group, there was interaction effect with abnormities of vagina cleanness, β-glucuronidase and neuraminidase on CIN1 and with abnormities of vagina cleanness, PH, H_2_O_2_, β-glucuronidase, neuraminidase and leukocyte esterase on CIN2/3 (**Table 5**).

## Discussion

The vaginal micro-environment plays an important role in reproductive health. The vaginal micro-environment disorder is closed to sexually transmitted diseases, bacterial vaginitis, and so on. But there is no clear definition about vaginal micro-environment disorder up to now. Vaginal PH value, H_2_O_2_, vagina cleanness, β-glucuronidase, coagulase, neuraminidase and leukocyte esterase, which could comprehensively reflect vaginal micro-ecology from vaginal acidity and alkalinity, Lactobacillus function, microbial metabolites and inflammatory reaction, are generally referred as vaginal micro-environmental factors to evaluate the vaginal micro-ecology in clinical practice 10,11.

*Lactobacillus,* which is dominant bacteria in vagina, plays an important role in maintaining vaginal microenvironment. Some research showed that *Lactobacillus* could defend against pathogens and sexually transmitted infections through maintenance of a hostile PH and production of species metabolites, bacteriocin 19, while increase of PH value and decrease of H_2_O_2_ in vagina are closely related to the occurrence of vaginitis 20. Incidence of precancerous lesions increased significantly in individuals with poor vagina cleanness than normal population 21. In the study, we found that the abnormal rates of H_2_O_2_, PH and vagina cleanness gradually increased with the severity of cervical neoplasm, and the abnormity of H_2_O_2_, PH and vagina cleanness could increase the risk of CIN. It suggested that H_2_O_2_, PH and vagina cleanness might be used as indicators to predict the progress of CIN.

In recent years, the combined detection of bacterial enzymes, such as, β-glucuronidase, coagulase, neuraminidase and leukocyte esterase, has been applied widely to evaluate the vaginal micro-ecology. Neuraminidase is closely related to *Gardnerella* infection 22. *Gardnerella* could utilize the complement regulatory molecule CD59 to activate the epithelial p38-mitogen-activated protein kinase pathway in human epithelial cells, leading to cell death, *Lactobacillus* reduction and PH value elevation 23. High expression of neuraminidase NEU3 in cancer cells leads to protection against programmed cell death, while in contrast, decreased NEU3 induces apoptosis, implying a critical role of NEU3 in the survival of cancer cells 24. These studies indicated that vaginal neuraminidase may be a potential target for cervical cancer diagnosis and therapy. The results in our study showed that neuraminidase increased with the severity of CIN, suggesting that neuraminidase could be used as a potential biomarker in cervical lesions screening.

β-glucuronidase has been found to be associated with *Escherichia coli* 25. Microbiome communities with abundant* L. crispatus* likely contribute to prevent *Escherichia coli* colonization and inhibit *Escherichia coli* activity in vagina 26. β-glucuronidase may promote virulence by destroying the protective mucosa barrier, thus increasing susceptibility to HPV 15. Therefore, β-glucuronidase has an important warning role in CIN progression.

*Enterococci* and *Staphylococcus aureus* in the vagina produce coagulase 26. *Enterococcus* can produce volgamycin, which effectively inhibit the growth of spoilage organism, however, the coagulase produced by *Staphylococcus aureus* will deposit fibrin on the surface of the cells, inhibiting the phagocytosis of phagocytic cells 27. Seidi et al 28 took advantage of the unique coagulation properties of *Staphylococcus coagulase* and genetically engineered it to generate a new fusion protein with novel anti-cancer properties. Our study showed that abnormal rate of coagulase changes along cervical lesions progress. The coagulase alteration which reflects ratio of *Enterococcus* to *Staphylococcus aureus* may provide meaningful clues to the association between vaginal bacterial and CIN.

Vaginal microbial diversity could reduce ability of the immune system to clear HPV 29, 30. Some studies also reported that women with high vaginal microbial diversity were most likely to have persistent HPV infection 31, 32 and CIN 33. In this study, we explored the associations between vaginal micro-environment factors and CIN in different HPV16 infection states. We found that the abnormal rates and risks of H_2_O_2_, cleanness, β-glucuronidase, neuraminidase were higher in CIN1 and CIN2/3 in HPV16 positive group. Hence, to detect vaginal micro-environment factors would be of great significance to prevent the progress of CIN in HPV16 positive women. Anita Mitra et al 34 identified *Garderella* and* L. iners* as the most high-risk combination for the development of CIN with an odds ratio of 34.1 in HR-HPV positive women compared to HR-HPV negative women. We also found that women with both vaginal micro-environment factors abnormality and HPV16 positive had a significantly higher risk for developing CIN. It was suggested that there might be synergistic effect between vaginal micro-environment factors abnormality and HPV16 infection in the progression of CIN.

A recent study demonstrated that *Gardnerella*, *Atopobium*, *Prevotella*, *Sneathia* were significantly enriched, whereas *Lactobacillus spp.* was underrepresented in cervical cancer group as well as in CIN group 12. However, the interaction of multiple vaginal micro-environment factors that secrete these pathogens was not defined on the occurrence of CIN. Our results from GMDR analysis showed the synergistic effect between abnormities of vagina cleanness, H_2_O_2_, β-glucuronidase and neuraminidase played an important role on CIN, and the role would be strengthened in women with HPV16 infection added with abnormities of PH and leukocyte esterase on CIN2/3. Lactic acid preferentially lyses bacteria other than *Lactobacillus* species 35, 36 and causes bacterial cell death by acidifying the cytosol, disrupting intracellular function 37, increasing the permeability of the cell membrane to H_2_O_2_, diacetyl et al. *Gardnerella* secretes neuraminidase that degrades vaginal mucus by cleaving sialic acid from the glycoproteins, increasing the risk of cervical disease 37. A synergistic effect between *Gardnerella* and *Prevotella* was found in BV women 38. In conclusion, our results suggested that vaginal micro-environment disorder could increase the risk of CIN, especially, the abnormality of H_2_O_2_, cleanness, β-glucuronidase and neuraminidase was of great significance to CIN regardless of HPV16 infection. There was interaction effect with abnormities of vagina cleanness, β-glucuronidase and neuraminidase on CIN regardless of HPV16 infection, particularly, with more vaginal micro-environment factors participating interaction effect in HPV16 positive group than HPV16 negative group. Although our study can't prove the causal association, we have found some valuable clues. Certainly, prospective cohort studies and experiment studies will be needed to provide powerful evidence in our future study.

## Figures and Tables

**Table 1:** Socio-demographic characteristics and related factors among cervical lesion groups

Variables	NC	CIN1	CIN2/3	*P*-value^*^
**Occupation**				0.041
Peasant	156(25.0)	93(30.7)	33(35.5)	
Others	467(75.0)	210(69.3)	60(64.5)	
**Passive smoking**				0.001
Yes	360(57.8)	212(70.0)	63(67.7)	
No	263(42.2)	91(30.0)	30(32.3)	
**Frequency of vulva cleaning**				0.032
≤1 time/month	20(3.2)	19(6.3)	9(9.7)	
3 times/week-1 time/month	263(42.2)	118(38.9)	39(41.9)	
≥3 times/week	340(54.6)	166(54.8)	45(48.4)	
**Frequency of underwear washing**				0.003
≤1 time/month	10(1.6)	10(3.3)	8(8.6)	
3 times/week-1 time/month	240(38.5)	108(35.6)	29(31.2)	
≥3 times/week	373(59.9)	185(61.1)	56(60.2)	
**Washing vulva after sexual intercourse**				0.027
Yes	470(75.4)	250(82.5)	77(83.8)	
No	153(24.6)	53(17.5)	16(17.2)	
**Contraception history**				0.023
Yes	549(88.1)	276(91.1)	75(80.6)	
No	74(11.9)	27(8.9)	18(19.4)	

^*^ *P -*values for differences between groups were obtained from the chi-square test.

**Table 2:** Eigenvalues of the related matrices

Component	Eigenvalues	Contribution rate (%)	Cumulative contribution rate (%)
1	1.479	26.467	24.647
2	1.126	18.771	43.418
3	1.049	17.478	60.897
4	0.966	16.094	76.991
5	0.838	13.959	90.949
6	0.543	9.051	100.00

**Table 3:** Orthogonal rotation of the factor load

Factors	FAC1	FAC2	FAC3
Occupation	-0.083	0.740*	0.249
Passive smoking	0.096	-0.034	0.839*
Frequency of vulva cleaning	0.849*	-0.008	0.109
Frequency of underwear washing	0.816*	0.071	-0.200
Washing the vulva after sexual intercourse	-0.142	-0.751*	0.196
Contraception history	-0.112	0.046	0.492*

* Factors corresponding to latent variables (FAC1, FAC2, FAC3).

**Table 4:** Association between vaginal micro-environment factors change and CIN in different HPV16 infection groups

Factors	HPV16 negative group				HPV16 positive group		
	Group	Abnormal persons (%)	*aOR^#^* (95%*CI*)		Group	Abnormal persons (%)	*aOR^#^* (95%*CI*)
**H_2_O_2_**	NC	395(72.1)	1.00		NC	57(76.0)	1.00
	CIN1	184(77.0)	1.34(0.94-1.92)		CIN1	56(87.5)	2.03(1.38-2.98)
	CIN2/3	45(86.5)	2.54(1.12-5.76)		CIN2/3	38(92.7)	4.10(1.60-10.52)
	*χ*^2^=6.41,*P*=0.041; *χ*^2^*_trend_* =6.10,*P*=0.014				*χ*^2^=6.42,*P=*0.040; *χ*^2^*_trend_* =6.09,*P=*0.014		
**PH**	NC	396(72.3)	1.00		NC	53(70.7)	1.00
	CIN1	179(74.9)	1.15(0.81-1.64)		CIN1	52(81.3)	1.78(0.79-3.99)
	CIN2/3	41(78.8)	1.43(0.71-2.85)		CIN2/3	30(90.2)	3.69(1.16-11.74)
	*χ*^2^=1.43,*P=*0.490; *χ*^2^*_trend_* =1.40,*P=*0.236				*χ*^2^=6.43,*P=*0.040; *χ*^2^*_trend_* =6.38,*P=*0.012		
**Vagina cleanness**	NC	213(35.9)	1.00		NC	29(38.7)	1.00
	CIN1	121(50.6)	1.54(1.13-2.10)		CIN1	36(56.3)	2.05(1.03-4.05)
	CIN2/3	31(59.6)	2.26(1.26-4.04)		CIN2/3	29(70.7)	3.84(1.67-8.83)
	*χ*^2^=15.22,*P<*0.001; *χ*^2^*_trend_* =15.12,*P<*0.001				*χ*^2^=11.57,*P=*0.003; *χ*^2^*_trend_* =11.47,*P=*0.00		
**β-glucuronidase**	NC	61(10.7)	1.00		NC	8(11.1)	1.00
	CIN1	38(15.9)	1.44(0.93-2.24)		CIN1	17(26.6)	2.92(1.15-7.38)
	CIN2/3	17(32.7)	3.73(1.97-7.08)		CIN2/3	16(39.0)	4.89(1.83-13.09)
	*χ*^2^=19.74,*P<*0.001; *χ*^2^*_trend_* =16.51,*P<*0.001				*χ*^2^=12.93,*P=*0.002;*χ*^2^*_trend_* =12.79,*P<*0.001		
**coagulase**	NC	104(19.0)	1.00		NC	20(26.7)	1.00
	CIN1	33(13.8)	0.65(0.42-1.01)		CIN1	10(15.6)	0.51(0.22-1.19)
	CIN2/3	14(26.9)	1.59(0.82-3.06)		CIN2/3	15(36.6)	1.59(0.70-3.59)
	*χ*^2^=6.00,*P*=0.050; *χ*^2^*_trend_* =0.01,*P*=0.910				*χ*^2^=6.05,*P=*0.048; *χ*^2^*_trend_* =0.59, *P=*0.441		
**neuraminidase**	NC	125(22.8)	1.00		NC	17(22.7)	1.00
	CIN1	72(30.1)	1.41(0.98-1.99)		CIN1	26(40.6)	2.34(1.11-4.93)
	CIN2/3	24(46.2)	2.91(1.62-5.22)		CIN2/3	26(63.4)	5.64(2.40-13.25)
	*χ*^2^=15.81,*P<*0.001; *χ*^2^*_trend_* =14.75,*P<*0.001				*χ*^2^=18.84,*P<*0.001;*χ*^2^*_trend_* =18.64,*P<*0.001		
**leukocyte esterase**	NC	233(42.5)	1.00		NC	29(38.7)	1.00
	CIN1	117(49.0)	1.33(0.98-1.82)		CIN1	33(51.6)	1.67(0.84-3.32)
	CIN2/3	25(48.1)	1.26(0.71-2.23)		CIN2/3	26(63.4)	3.02(1.33-6.82)
	*χ*^2^=3.05,*P*=0.218; *χ*^2^*_trend_* =2.47,*P*=0.116				*χ*^2^=6.78,*P=*0.034; *χ*^2^*_trend_* =6.74,*P=*0.009		

*^#^*Adjusted by FAC1, FAC2 and FAC3

**Table 5:** Interaction of vaginal micro-environment factors on CIN by GMDR analysis

Group	Model	TBA	*P*	CVC
**HPV16 negative**				
CIN1	A1	0.4763	0.9453	6/10
	A1/ A3	0.6058	0.0510	10/10
	A1/A3/A4	0.5457	0.1719	7/10
	A1/A2/A3/A4	0.5570	0.0547	10/10
CIN2/3	A4	0.5926	0.1719	7/10
	A3/A4	0.6266	0.0547	8/10
	A2/A3/A4	0.5950	0.0107	10/10
	A1/A2/A3/A4*	0.6394	0.0107	10/10
**HPV16 positive**				
CIN1	B1	0.4499	0.9893	5/10
	B1/B4	0.5180	0.0547	7/10
	B1/B4/B6*	0.5832	0.0107	10/10
	B2/B3/B5/B7	0.5110	0.3770	3/10
	B2/B3/B4/B5/B7	0.5132	0.3770	7/10
	B2/B3/B4/B5/B6/B7	0.5496	0.3770	5/10
	B1/B2/B3/B4/B5/B6/B7	0.5521	0.3770	10/10
CIN2/3	B6	0.6284	0.0107	10/10
	B4/B6	0.6266	0.0547	8/10
	B3/B6/B7	0.5547	0.1719	4/10
	B1/B3/B6/B7	0.5742	0.1719	4/10
	B1/B3/B4/B6/B7	0.6333	0.0547	8/10
	B1/B2/B3/B4/B6/B7*	0.6394	0.0107	10/10
	B1/B2/B3/B4/B5/B6/B7	0.6009	0.0107	10/10

A1-A4 represents vagina cleanness, H_2_O_2_, β-glucuronidase, neuraminidase. B1-B7 represents vagina cleanness, PH, H_2_O_2_, β-glucuronidase, coagulase, neuraminidase, and leukocyte esterase respectively. TBA represents testing balance accuracy. CVC represents cross validation consistency. *The best interaction model adjusted by FAC1, FAC2 and FAC3.
